# The family of 14‐3‐3 proteins and specifically 14‐3‐3σ are up‐regulated during the development of renal pathologies

**DOI:** 10.1111/jcmm.13691

**Published:** 2018-06-28

**Authors:** Myrto Rizou, Eleni A. Frangou, Filio Marineli, Niki Prakoura, Jerome Zoidakis, Harikleia Gakiopoulou, George Liapis, Panagiotis Kavvadas, Christos Chatziantoniou, Manousos Makridakis, Antonia Vlahou, John Boletis, Demetrios Vlahakos, Dimitrios Goumenos, Evgenios Daphnis, Christos Iatrou, Aristidis S. Charonis

**Affiliations:** ^1^ Biomedical Research Foundation of the Academy of Athens Athens Greece; ^2^ Laikon University Hospital, Nephrology Clinic, Medical School National and Kapodistrian University of Athens Athens Greece; ^3^ First Department of Pathology Medical School National and Kapodistrian University of Athens, and Laikon Hospital Athens Greece; ^4^ INSERM UMRS 1155 Tenon Hospital Paris France; ^5^ Division of Nephrology Attikon University Hospital National and Kapodistrian University of Athens Medical School Athens Greece; ^6^ Department of Nephrology Medical School of Patras University Hospital of Patras Rio Greece; ^7^ Medical School of the University of Crete University Hospital of Iraklion Iraklion Greece; ^8^ Center for Nephrology “G. Papadakis” General Hospital of Nikaia‐Piraeus Athens Greece

**Keywords:** 14‐3‐3 proteins, 14‐3‐3σ, calreticulin, HIF1α, Hypoxia, renal pathologies

## Abstract

Chronic kidney disease, the end result of most renal and some systemic diseases, is a common condition where renal function is compromised due to fibrosis. During renal fibrosis, calreticulin, a multifunctional chaperone of the endoplasmic reticulum (ER) is up‐regulated in tubular epithelial cells (TECs) both in vitro and in vivo. Proteomic analysis of cultured TECs overexpressing calreticulin led to the identification of the family of 14‐3‐3 proteins as key proteins overexpressed as well. Furthermore, an increased expression in the majority of 14‐3‐3 family members was observed in 3 different animal models of renal pathologies: the unilateral ureteric obstruction, the nephrotoxic serum administration and the ischaemia‐reperfusion. In all these models, the 14‐3‐3σ isoform (also known as stratifin) was predominantly overexpressed. As in all these models ischaemia is a common denominator, we showed that the ischaemia‐induced transcription factor HIF1α is specifically associated with the promoter region of the 14‐3‐3σ gene. Finally, we evaluated the expression of the family of 14‐3‐3 proteins and specifically 14‐3‐3σ in biopsies from IgA nephropathy and membranous nephropathy patients. These results propose an involvement of 14‐3‐3σ in renal pathology and provide evidence for the first time that hypoxia may be responsible for its altered expression.

## 1 INTRODUCTION

Chronic kidney disease (CKD) affects over 10% of the adult population and this percentage is expected to increase due to extended lifetime and the epidemics of hypertension and diabetes.^1^ CKD is characterized by a progressive loss of renal function, which, irrespective of the cause (primary renal diseases or systemic diseases) has as a common pathological manifestation in the development of fibrosis. Renal fibrosis is a complicated, multi‐factorial process, where many cellular and molecular mediators are involved and often leads to glomerulosclerosis and tubular atrophy.^2^, ^3^, ^4^, ^5^

In a discovery‐driven approach, our laboratory has used proteomic analysis in the well‐established unilateral ureteral obstruction (UUO) rodent model of renal fibrosis. Among the proteins that were found strongly up‐regulated, we have identified calreticulin, a multifunctional ER Ca^2+^ binding protein which also functions as a chaperone. We have shown that its overexpression in vitro induced a strong profibrotic phenotype in tubular epithelial cells (TECs) and inversely, its down‐regulation in calreticulin heterozygous mice preserved kidneys from developing fibrosis.^6^, ^7^

To further explore the interrelation between calreticulin and fibrosis, we studied cellular phenotype modifications of TECs induced by calreticulin overexpression. For this purpose, we investigated the alterations in proteome of cultured human proximal renal TECs stably overexpressing calreticulin. The differentially expressed proteins were organized into protein‐protein interaction networks that pointed towards the central role of 14‐3‐3 proteins, a family of acidic, low molecular weight, conserved proteins, expressed in all eukaryotic cells. In humans, there are 7 isoforms denoted as β, ε, γ, η, τ, ζ and σ that are coded by 7 distinct genes. 14‐3‐3 proteins play crucial role in a wide range of cellular activities, including cell proliferation, protein trafficking, DNA replication, cell apoptosis and survival.^8^, ^9^ This family of proteins has been involved in numerous neurological disorders while some members of the family such as 14‐3‐3ζ, 14‐3‐3γ and 14‐3‐3σ are associated with a variety of human cancers.^10^, ^11^ However, their role in the process of renal pathologies was not studied so far.

In this report, we focus on the involvement of the 14‐3‐3 family of proteins in renal pathology. To this end, we have first validated that the 14‐3‐3 proteins are up‐regulated following UUO. Subsequently, we studied the expression of this family in 2 other animal models of renal pathology, those of nephrotoxic serum administration (NTS) and ischaemia‐reperfusion injury (IR) and we found that the 14‐3‐3σ member of this family, also known as stratifin, showed the highest up‐regulation. Since a common characteristic of the above animal models is the underlying ischaemia,^12^, ^13^ we showed that the ischaemia‐induced transcription factor HIF1α^14^ is specifically associated with the promoter region of the 14‐3‐3σ gene. Finally, we evaluated the expression of the family of 14‐3‐3 proteins and specifically 14‐3‐3σ in biopsies from IgA nephropathy and membranous nephropathy patients.

Overall, our findings strongly support an involvement of the family of 14‐3‐3 proteins and especially 14‐3‐3σ in renal pathology possibly through its regulation by HIF1α.

## 2 MATERIALS AND METHODS

### 2.1 Proteomic analysis

Two independent clones of human proximal TECs (HK‐2 cell line) stably overexpressing calreticulin in similar levels and 2 control clones were studied with proteomic analysis. From each clone, 2 biological and 2 technical replicates were analysed with 2D electrophoresis. From each sample, 100 μg of protein was applied onto immobilized pH gradient strips of non‐linear p*I* 3‐10 (BioRad). Isoelectric focusing was performed in BIORAD PROTEAN IEF cell. Strips were reduced and alkylated, placed onto 12% acrylamide gels and electrophorised at 180 V.^15^ 2D gels were incubated in fixation buffer (30% methanol, 10% acetic acid), stained with Coomassie Colloidal Blue and destained with double distilled water. Gels were scanned with GS‐800 calibrated densitometer and analysed with PDQuest 8 image processing software (BioRad). Normalization of spot intensities was conducted according to total OD in the gel. Spots exhibiting quantitative differences between the gels were excised and placed into 96‐well plates. Spots were distained and following reduction and alkylation they were dried in MAXI DRY PLUS, and digested with 3 μL trypsin (10 ng/μL) overnight. Peptides were extracted with 10 μL 50% ACN, 0.1% TFA. Peptide mixtures were analysed with a matrix‐assisted laser desorption/ionization‐time‐of flight/time‐of‐flight (MALDI‐TOF/TOF) mass spectrometer (Ultralfex II MALDITOF/TOF‐MS, Brucker Daltonics, Bremen, Germany). Peaklists were created using Flexanalysis v2.2 software (BruckerDaltonics). Peptide matching and protein searches were performed automatically by the use of MASCOT Software (Matrix Sciences, London, UK). For peptide identification, monoisotopic masses were used and a mass tolerance of 0.0025% (25 ppm) was allowed. Cysteine carbamidomethylation and methionine oxidation were set as fixed and variable modifications, respectively. One miscleavage site was allowed. The peptide masses were compared with the theoretical peptide masses of all available proteins from Homo sapiens using Swiss‐Prot database. The probability score with *P* < .05 identified by the software was used as the criterion for the affirmative protein identification. The identified proteins were categorized according to their function and gene ontology using UniProt database (Universal Protein Resource).^16^ Protein‐protein interaction networks were created with the Ingenuity Pathway Analysis software (IPA)^17^ using the accession number of the identified differentially expressed proteins.

### 2.2 Cell culture

Human proximal TECs (HK‐2) were purchased from ATCC (Manassas, VA) and grown in 1:1 Dulbecco's modified Eagle's medium 4.5 g/L glucose and F‐12 supplemented with 10% foetal bovine serum (FBS), 100 mg/mL penicillin‐streptomycin and 2 mmol/L L‐glutamine. Calreticulin overexpressing HK‐2 cell lines were generated as described in Ref. 6 Primary renal TECs were isolated from kidneys of 8‐ to 12‐week‐old male C57BL/6 mice according to a protocol modified from Ref. 18 Briefly, whole kidneys were decapsulated, finely minced into small pieces using a scalpel and digested in 1 mg/mL collagenase I for 3 minutes at 37°C. The digested tissue was loaded on a 70‐μm filter, smashed using a syringe plunger and flushed with complete culture medium (RPMI 1640, supplemented with 10% FBS and 100 U/mL penicillin/streptomycin) in a falcon tube. Next, the cell mixture containing tubules and glomeruli was gently shaken and passed through a 40‐μm filter. The glomeruli were blocked on the filter while the tubules passed through. The tubular cell mixture was centrifuged, re‐dissolved in complete culture medium and seeded in plates. Cells grew for up to 1 week with culture medium being replaced every 3 days. For the induction of hypoxia, cells were incubated in media containing 2% FBS for 2 hours and then subjected to hypoxia by mineral oil overlay for 1 hour. Control cells were incubated with complete medium.

### 2.3 UUO mouse model

Eight‐ to 12‐week‐old male C57BL/6 mice were supplied from the colony of out Center of Experimental Surgery. For the UUO model, mice were anesthetized via face mask delivering sevoflurane and underwent ligation of the right ureter. Control animals (sham operated) were manipulated similarly but not ligated.^19^ Mice were euthanized 2 or 8 days after surgery and renal tissue was collected for subsequent analyses.

All aspects of animal experimentation were performed in adherence to the NIH and the European Union Guide for the Care and Use of Laboratory Animals and were approved by the Institutional Review Board and the Animal Experimental Committee of the Biomedical Research Foundation of the Academy of Athens (Permit Number: K5761).

### 2.4 Other mouse models

For the renal ischaemia‐reperfusion (IR) model, 8‐ to 10‐week‐old FVB male mice were used (18). The mice were anaesthetized with intraperitoneal injection of ketamine (100 mg/kg)/xylazine (10 mg/kg) and subjected to right kidney nephrectomy. Right kidneys were used as controls. The left renal artery was clamped for 35 minutes of warm ischemia at 37°C followed by 24 hours or 72 hours of reperfusion. Renal tissue was collected for further analysis.

The nephrotoxic serum‐induced glomerulonephritis model (NTS) was performed in 8‐ to 10‐week‐old SV129 male mice. Crescentic glomerulonephritis was induced by intravenous injections of 12 μL NTS per g bodyweight over 2 consecutive days while control mice were injected with PBS. Mice were euthanized 9 days after first injection and renal tissues were collected.^20^

All aspects of animal experimentation were performed in adherence to the NIH and the European Union Guide for the Care and Use of Laboratory Animals and were approved by the National Institute for Health and Medical Research (Institut National de la Santé et de la Recherché Médicale).

### 2.5 RNA isolation, cDNA synthesis and Quantitative Real‐Time PCR analysis

Total RNA was isolated from cultured cells and mouse kidney tissue using TRI reagent (Life Technologies, Carlsbad, CA) according to the manufacturer's instructions. Residual genomic DNA was removed by treatment with RQ1 DNase (Promega Corp., Madison, WI). RNA was transcribed to cDNA using ImProm‐II reverse transcriptase (Promega Corp., Madison, WI). Quantitative real‐time PCR analysis (RT‐qPCR) was performed in Lightcycler 96 (Roche) using Platinum TaqDNA polymerase (Invitrogen Corp., Carlsbad, CA), three‐step standard cycling conditions and sequence‐specific primers (Tables S1 and S2). Cycle conditions consisted of a pre‐incubation step at 95°C for 10 minutes, 45 cycles of 95°C for 10 seconds, 60°C for 15 seconds (plate reading), 72°C for 15 seconds. Melting curve analysis was performed, with measurements taken every 1°C from 65°C to 95°C to verify that a single product was amplified. For quantitative analysis, experimental genes were normalized to GAPDH or HPRT gene expression using the ΔΔCT method. Experiments were performed in triplicate and repeated 3 times.

### 2.6 Western blot analysis

Proteins from cells and tissue samples were extracted in RIPA buffer [50 mmol/L Tris (pH 7.4), 1% NP‐40, 0.25% deoxycholate (DOC), 150 mmol/L NaCl, 1 mmol/L Na_2_EDTA, 1 mmol/L phenylmethylsulfonyl fluoride supplemented with cocktail protease inhibitors (Sigma‐Aldrich)] and measured for protein concentration using the Bradford method. Equal protein amounts were subjected to electrophoresis in 12% polyacrylamide gels and transferred to nitrocellulose membranes. Membranes were blocked with 5% skimmed milk in 1 × TBS/Tween for 1 hour at RT and then incubated with primary antibodies against calreticulin (06‐661; Merck Millipore), pan 14‐3‐3 (sc‐629; Santa Cruz Biotechnology) and 14‐3‐3σ (ab14123; Abcam). The bound antibodies were labelled with horseradish peroxidase‐conjugated secondary antibodies (Sigma‐Aldrich) and detected with ECL (Perkin Elmer) detection system. Western blot images were analysed and quantified with ImageJ software (v1.48, NIH, Bethesda, MD). Gapdh (G9545; Sigma) was used as loading control.

### 2.7 Chromatin Immunoprecipitation

ChIP was performed as previously described in Ref. 21 in kidneys from C57BL/6 mice that have been subjected to UUO for 2 or 8 days and to sham‐operated animals. More specifically, immunoprecipitation was carried out with an antibody against HIF1α (NB100‐105; Novus Biologicals) while normal rabbit IgG (sc‐2027; Santa Cruz Biotechnology) was used as control. Precipitated complexes were detected with RT‐qPCR using the Platinum SYBR Green qPCR supermix‐UDG kit (Invitrogen; #11733‐046) and specific primers for selected sites in mouse 14‐3‐3σ promoter (Table S3). Primers were designed based on bioinformatics analysis of 14‐3‐3σ promoter sequences retrieved from Genomatix Suite Tools.^16^ Results were expressed as percentage of the input. Experiments were performed in triplicate and repeated 3 times.

### 2.8 Immunohistochemistry

Immunohistochemical detection of 14‐3‐3 proteins was performed in formalin‐fixed paraffin‐embedded mouse kidney tissue of archived kidney biopsy material from patients suffering from IgA nephropathy (n = 13) and membranous nephropathy (n = 10). For the studies in human tissues, healthy renal tissue from areas that were away from renal carcinoma was used as control (n = 5). The use of human kidney biopsy material was approved by the ethics committee of Medical School of National and Kapodistrian University of Athens. Sections were de‐paraffinized and rehydrated. Endogenous peroxidase was inactivated with 3% H_2_O_2_ in methanol for 30 minutes at room temperature in the dark. Sections were treated for antigen retrieval with 10 mmol/L sodium citrate (pH 6.0) at 96°C for 10 minutes, blocked with 10% FBS, 0.1% Triton X‐100 solution and incubated with primary antibodies against pan 14‐3‐3 (sc‐629; Santa Cruz Biotechnology, 1:4000) or 14‐3‐3σ (sc‐7683; Santa Cruz Biotechnology, 1:50) overnight at 4°C. For negative controls, sections were incubated with non‐specific rabbit or goat IgG (Sigma‐Aldrich). Finally, tissue sections were incubated with anti‐rabbit or anti‐goat HRP secondary antibody (Sigma‐Aldrich, 1:500) for 1 hour at room temperature. Detection reactions were developed with 3.3‐diaminobenzidine (SK‐4100, Vector Laboratories, Inc. Burlingame, CA) and slides were counterstained with haematoxylin (RRSP 65‐F, Atom Scientific, UK). Sections were dehydrated in graded alcohols and xylene and mounted in DPX. At least 5 non‐overlapping images were obtained using a Leica DM LS2microscope and a DFC 500 camera.

### 2.9 Statistical analysis

Results are presented as mean ± SEM. Data were analysed using one‐way ANOVA followed by Fischer test or the Mann‐Whitney U test. Values of *P* < .05 were considered significant.

## 3 RESULTS

### 3.1 Proteomic analysis reveals alterations in proteome of TECs stably overexpressing calreticulin

**Figure 1:**
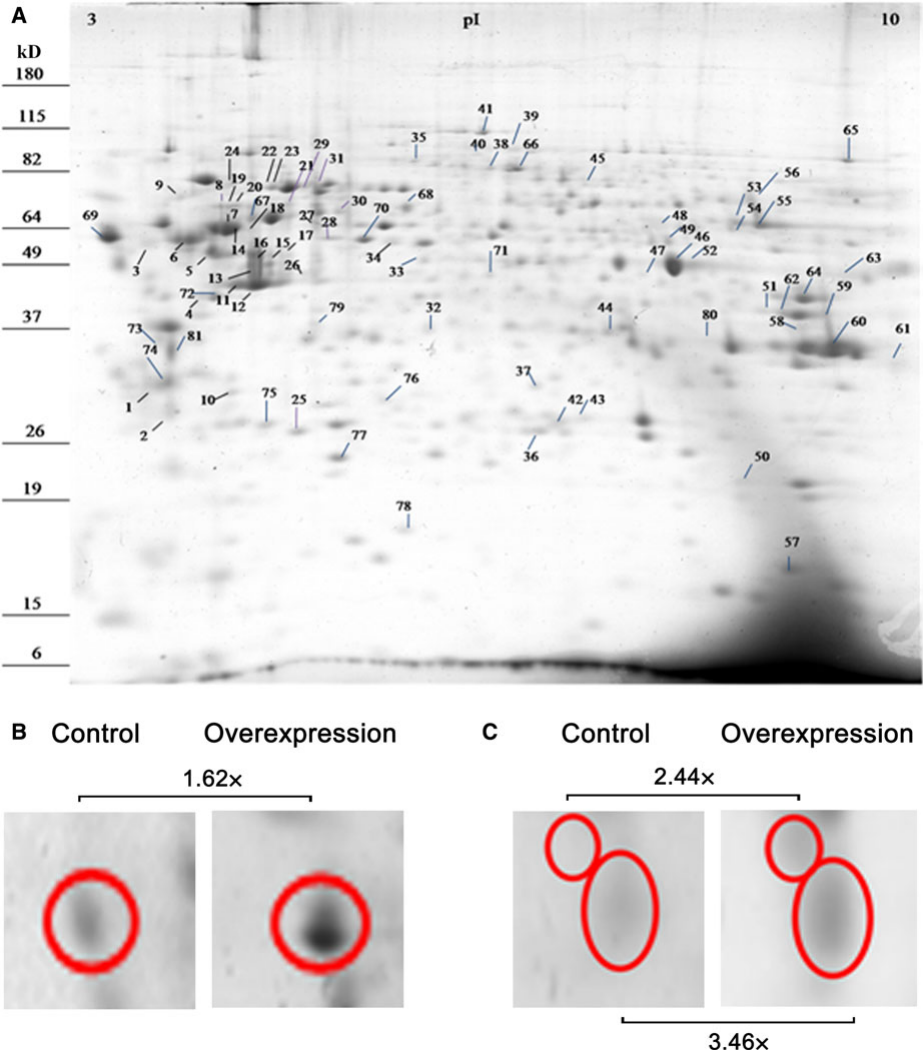
The proteomic profile of tubular epithelial cells that overexpress calreticulin is altered. A, Representative 2D gel image. Calreticulin was identified from spot number 69; 14‐3‐3 proteins were identified from spots 1 and 2. B, Spots corresponding to calreticulin in control and in calreticulin overexpressing cells with the corresponding fold‐changes. C, Spots corresponding to 14‐3‐3 proteins in control and in calreticulin overexpressing cells with the corresponding fold‐changes. The 2 circles represent different 14‐3‐3 isoforms migrating in slightly different positions in the 2D gels

To investigate how the cellular phenotype of renal TECs is altered during calreticulin overexpression, we performed proteomic analysis in 2 independent clones of human proximal TECs stably overexpressing calreticulin in similar levels and 2 control clones. Protein spots were matched in 2D electrophoresis gel images obtained from each clone and their intensity was compared after normalization (fold change > 2). From 81 differentially expressed spots, 74 differentially expressed proteins were identified (Figure 1A, Figure S1 and Table S4). Initially, calreticulin overexpression was confirmed in the cell lines studied (Figure 1B). The identified differentially expressed proteins were categorized using UniProt database according to their cellular function and gene ontology, and classified in 13 categories (Figure S2A). More specifically, calreticulin overexpression in TECs was associated with alterations in abundance in proteins involved in inflammation, cytoskeletal rearrangement, collagen production, extracellular matrix deposition, cellular metabolism, apoptosis, cellular redox homeostasis, protein folding and proteasomal degradation. Moreover, differences were observed in the levels of chaperones, heat shock proteins, cell cycle proteins and of proteins involved in mRNA alternative splicing. To identify protein‐to‐protein interaction networks among the differentially expressed proteins, the Ingenuity Pathway Analysis software was used. The proteins that are affected by calreticulin overexpression were organized in 4 networks; in three out of these 4 networks at least 1 member of the family of 14‐3‐3 proteins was present, indicating an involvement of this family in renal pathology (Figure 1C, Figure S2B). As 14‐3‐3 proteins have not been associated with renal diseases so far, they were selected for further study.

### 3.2 Confirmation of 14‐3‐3 proteins up‐regulation in vitro

**Figure 2:**
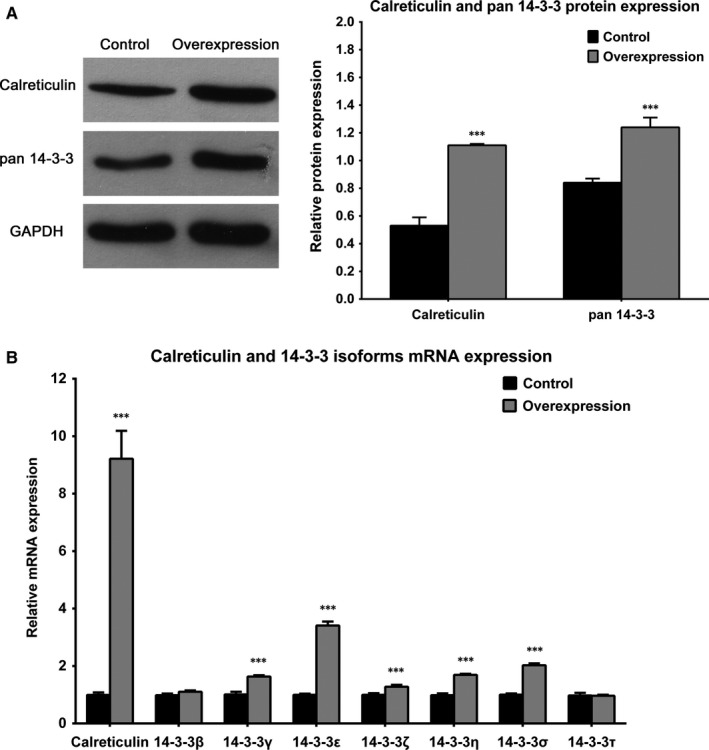
14‐3‐3 proteins are up‐regulated at protein and mRNA level in cultured tubular epithelial cells overexpressing calreticulin. A, Western blot analysis and corresponding quantification of protein abundance of calreticulin and 14‐3‐3 protein family in calreticulin overexpressing cells. n = 3 B, Relative mRNA abundance of calreticulin and 14‐3‐3 isoforms in calreticulin overexpressing cells. ***P < .001 vs control. n = 3

To validate the results of proteomic analysis, we performed western blot analysis in the studied cell lines using a pan antibody for all 7 isoforms of the 14‐3‐3 family of proteins. The results confirmed that in the presence of calreticulin overexpression, 14‐3‐3 proteins are up‐regulated (Figure 2A). To study each of the 14‐3‐3 family members separately, we performed RT‐qPCR with primers specifically designed for each of the seven 14‐3‐3 genes. The results showed that with the exception of 14‐3‐3β and 14‐3‐3τ isoforms, the mRNA coding for the other isoforms of 14‐3‐3 proteins is significantly up‐regulated upon calreticulin overexpression (Figure 2B). To support these results, RT‐qPCR data were also normalized against RPL32 and GUSB housekeeping genes (Figure S3 A and B).

### 3.3 14‐3‐3 proteins are up‐regulated in the UUO animal model

**Figure 3:**
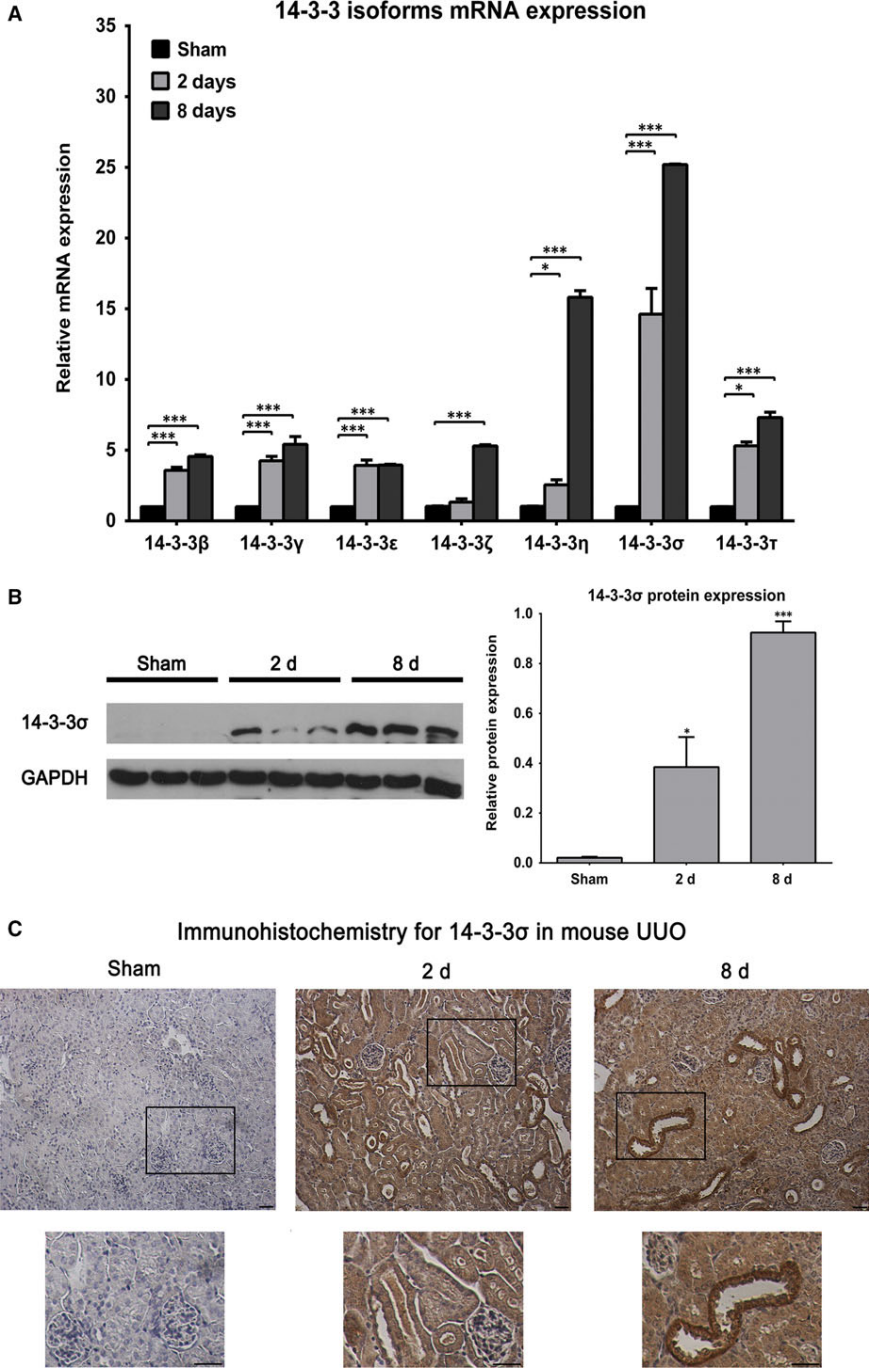
14‐3‐3 isoforms expression in the UUO model. A, Quantification of 14‐3‐3 isoforms expression levels by quantitative real‐time PCR analysis. Relative mRNA levels of 14‐3‐3 isoforms and especially those of 14‐3‐3σ showed significant increase at 2‐ and 8‐day ligation intervals compared to sham‐operated animals. B, Western blot analysis and corresponding quantification of 14‐3‐3σ isoform in sham operated, 2‐day ligated and 8‐day ligated animals. C, Localization of 14‐3‐3σ in mouse kidney sections from sham operated, 2‐day ligated and 8‐day ligated animals. As seen in the upper panel of sections, the expression of 14‐3‐3σ is increased during the progression of renal disease, whereas in lower panel of sections in increased magnification it is showed that 14‐3‐3σ is mainly expressed in the distal tubules of renal parenchyma. Proximal tubules exhibited weaker staining and glomeruli showed no significant staining. Scale bars. 100 μm. *P < .05; ***P < .001 vs control. n = 3 per group

To study the expression of 14‐3‐3 in vivo during the development of obstructive nephropathy in the UUO model, RT‐qPCR experiments were performed in samples of sham operated, 2‐and 8‐day ligated animals. These intervals were selected because at 2 days biochemical alterations have been established although fibrotic tissue has not been accumulated in the renal parenchyma of the ligated ureter, whereas at 8 days, extensive accumulation of fibrotic tissue is observed.^7^ The mRNA abundance of all 7 isoforms of 14‐3‐3 proteins was gradually increased between 2 and 8 days after ureteric obstruction. Of those, two had remarkably higher expression: the mRNA of 14‐3‐3η isoform exhibited a 15‐fold increase in 8‐day post‐ligation, whereas the mRNA of 14‐3‐3σ isoform showed a 14‐fold increase at 2 days after ligation and a 25‐fold increase at 8 days after ligation (Figure 3A). These results were also confirmed by data originated from whole‐transcriptome analysis of UUO mouse model that was performed in our laboratory.^22^ In particular, the 14‐3‐3σ isoform displayed a 2.70‐fold change at 2 days and a 10.96‐fold change at 8 days after ligation compared to sham samples.

Based on the above results, 14‐3‐3σ was studied next at the protein level both biochemically and morphologically. Western blot analysis, using an antibody specific for this isoform, revealed an increase in protein abundance that is in accordance with the transcriptional up‐regulation (Figure 3B). Immunohistochemistry studies further confirmed this finding. As shown in Figure 3C, in sham‐operated animals 14‐3‐3σ protein was minimally expressed in the cytoplasm of TECs; in 2 days post‐ureteric ligation, it was found substantially overexpressed in the cytoplasm of all TECs, whereas in 8‐day post‐ureteric ligation, its expression was accumulated mainly in distal tubules.

### 3.4 14‐3‐3 proteins are up‐regulated in other animal models of kidney diseases

**Figure 4:**
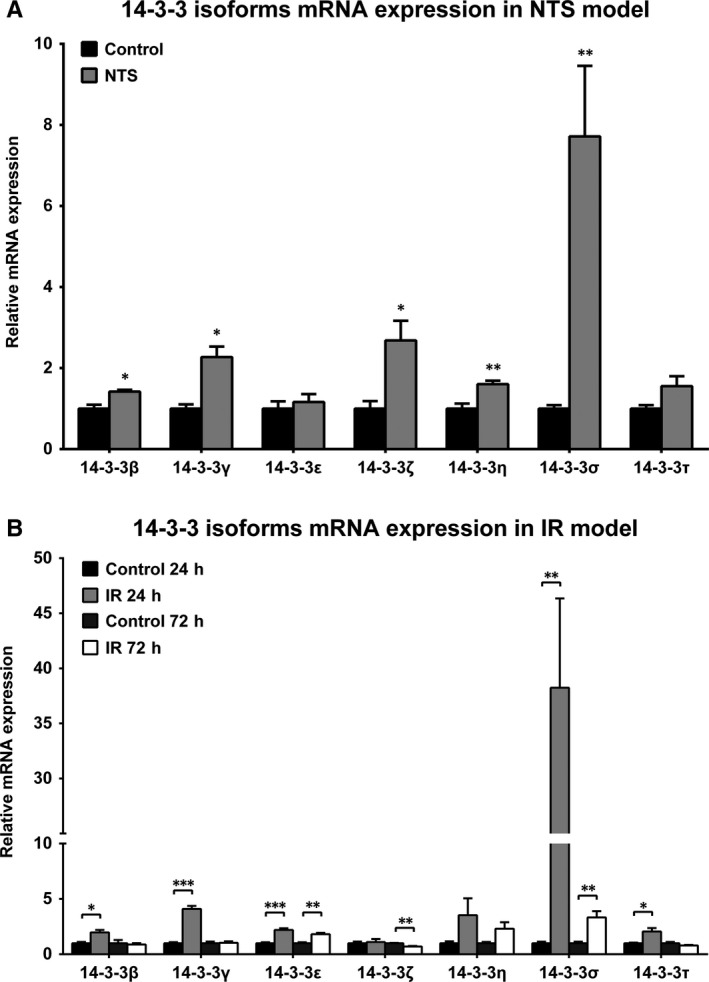
14‐3‐3 isoforms expression profile in other animal models of kidney diseases showed high similarity with the UUO model. A, Relative mRNA abundance of 14‐3‐3 isoforms in nephrotoxic serum‐induced glomerulonephritis (NTS) mouse model. B, Relative mRNA abundance of 14‐3‐3 isoforms in ischaemia‐reperfusion (IR) model. *P < .05; **P < .01; ***P < .001 vs control. n = 3 per group

Following the findings in the UUO model, the expression levels of the family of 14‐3‐3 proteins were further evaluated in 2 other animal models of nephropathy, the NTS model of glomerular injury and the IR model of acute renal injury. In agreement with the UUO model, the mRNA of most of the 7 isoforms of 14‐3‐3 proteins was found significantly up‐regulated in both NTS and IR mouse models. Moreover, in both models, 14‐3‐3σ exhibited the greatest level of change in expression compared to the other isoforms. Specifically, 14‐3‐3σ displayed a 6.7‐fold increase in NTS model, whereas it showed a 38‐fold increase 24 hours post‐ischaemia and a threefold increase 72 hours post‐ischaemia in the IR model (Figure 4A,B respectively). To further validate the results emerged from the initial analyses, in all animal models RT‐qPCR data were normalized against additional housekeeping genes including RPL32, GUSB and 18s (Figure S4, A‐F).

Taken together, the results from all animal models provide evidence that the up‐regulation of expression of the 14‐3‐3 family members and especially the 14‐3‐3σ isoform is a general finding during early stages of development of renal pathologies.

### 3.5 Hypoxia induces 14‐3‐3σ expression in tubular epithelial cells in vitro

**Figure 5:**
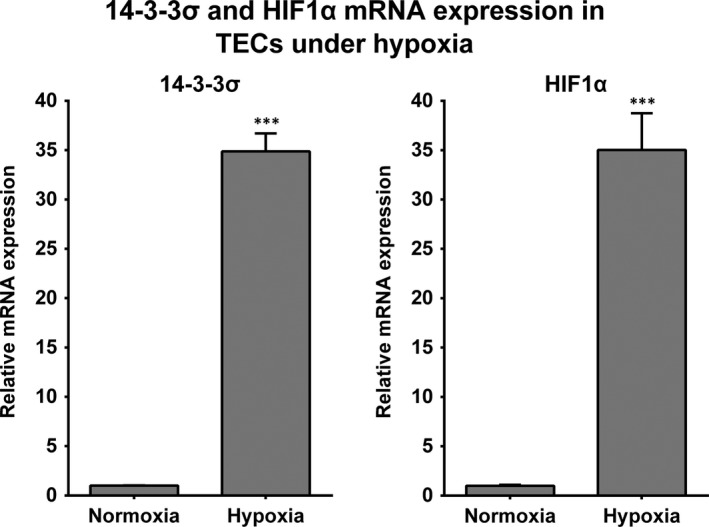
Hypoxia promotes 14‐3‐3σ expression in vitro. Relative mRNA abundance of 14‐3‐3σ and HIF1α, a key regulator of hypoxia, in mouse primary tubular epithelial cells under normoxic and hypoxic conditions. Hypoxia was induced by mineral oil overlay of the cells for 1 h. ***P < .001 vs normoxia. n = 3

Since a common characteristic at the early stage of renal disease in these animal models is the presence of hypoxia,^23^, ^24^ we aimed to investigate whether the expression of 14‐3‐3σ is affected under hypoxic conditions. For this purpose, we exposed to hypoxia primary cultures of mouse TECs. RT‐qPCR analysis demonstrated that 14‐3‐3σ expression is strongly induced after hypoxia. Consistently, HIF1α, a crucial transcription factor in hypoxia,^25^ was also found up‐regulated (Figure 5).

### 3.6 HIF1α interacts with the 14‐3‐3σ promoter in vivo in the UUO model

**Figure 6:**
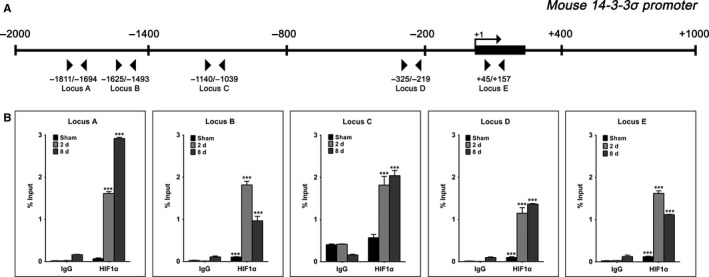
ChIP analysis performed in the UUO mouse model showed high enrichment of HIF1α transcription factor on 14‐3‐3σ promoter. A, Schematic representation of 14‐3‐3σ promoter showing the location of putative binding sites for HIF1α transcription factor as derived from bioinformatics analysis. B, Enrichment of HIF1α in different loci on 14‐3‐3σ promoter. Chromatin was prepared from kidneys of sham operated (Sham), 2‐day ligated and 8‐day ligated mice. The immunoprecipitated complexes were detected with RT‐qPCR using primers marked with arrows in A. ***P < .001 vs IgG. n = 3 per group

Based on the in vitro results and considering the fact that HIF1α has already been found up‐regulated in the UUO model,^12^ we evaluated the involvement of HIF1α in the transcriptional regulation of 14‐3‐3σ promoter in this model by performing ChIP assays. Using bioinformatics analysis, we found consensus motifs that are recognized by HIF1α in 14‐3‐3σ promoter (Figure 6A). The results shown in Figure 6B demonstrate that normal levels of HIF1α, in sham‐operated animals, are sufficient to allow its binding in loci B, D and E on 14‐3‐3σ promoter. Furthermore, it was shown that the binding pattern of HIF1α was different among its specific binding sites on 14‐3‐3σ promoter. In particular, the occupancy of the HIF1α putative acceptor sites was increased at 2 days ligation interval in all loci when compared to sham‐operated animals. A further increase was observed at 8‐day ligation interval at loci A, C and D, whereas at loci B and E, the enrichment on HIF1α was decreased at 8 days.

### 3.7 14‐3‐3 proteins are highly expressed in human membranous nephropathy and IgA nephropathy

**Figure 7:**
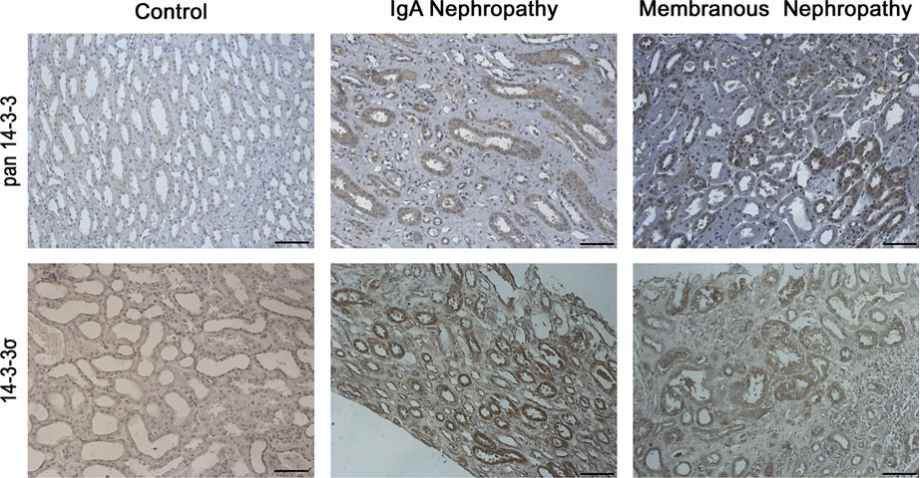
14‐3‐3 proteins are up‐regulated in kidneys of patients with IgA nephropathy and membranous nephropathy. Representative fields are shown following immunohistochemistry staining either with an antibody recognizing all members of the 14‐3‐3 family (upper panel) or with an antibody recognizing specifically the 14‐3‐3σ protein (lower panel). The intensity of the staining was significantly increased (*P* < .001 by Mann‐Whitney U test) in both pathologies compared to control samples, especially in the cytoplasm of distal tubular epithelial cells. In all cases, control tissue came from healthy kidney areas away from renal carcinoma. Scale bars, 100 μm

To investigate the expression of 14‐3‐3 proteins in human glomerulopathies, we analysed with immunohistochemistry the expression of 14‐3‐3 proteins in human kidney biopsies from 13 patients with IgA nephropathy and 10 patients with membranous nephropathy. The results from all sections studied establish that in both membranous and IgA nephropathy, there is a significant up‐regulation of 14‐3‐3 proteins when compared to healthy kidney areas in TECs (*P* < .001 by Mann‐Whitney U test) in 100% of the cases studied. These results were obtained using a pan antibody against all members of the 14‐3‐3 family (Figure 7, upper panel) as well as an antibody specific for the 14‐3‐3σ isoform (Figure 7, lower panel). Calreticulin was also up‐regulated in the same cells (data not shown). These results provide strong evidence that the expression of 14‐3‐3 proteins and especially 14‐3‐3σ is induced in IgA nephropathy and membranous nephropathy, in renal epithelial cells.

## 4 DISCUSSION

In view of the high incidence and the severity of CKD and considering the fact that symptoms are lacking or that are often non‐specific until CKD is advanced, it is imperative to understand the nature and the underlying mechanisms of this disease and to focus on the discovery of novel reliable biomarkers that reflect the process and the progression of the disease. Previous studies from our group have shown that calreticulin, an ER Ca^2+^ binding chaperone, is overexpressed in TECs during the progression of kidney disease in the UUO rodent model.^6^, ^7^ In the present study, we aimed to investigate the proteomic profile of calreticulin overexpressing TECs. Our findings from proteomic analysis indicated a critical role of the 14‐3‐3protein family in the development of renal pathology. These results were validated at the mRNA and the protein level in the mouse model of obstructive nephropathy and further confirmed in 2 other animal models, those of nephrotoxic serum nephropathy and ischaemia‐reperfusion injury. In these studies, the 14‐3‐3σ isoform (also known as stratifin) was detected as the member with the most altered expression. Since hypoxia is a key factor present in all 3 models examined, the involvement of the transcription factor HIF1α known as a master molecule in hypoxic events was examined in an in vitro system of hypoxia in TECs and in vivo in the UUO model and was detected specifically associated with the promoter of the 14‐3‐3σ gene. Finally, examination of biopsy material from IgA nephropathy and membranous nephropathy patients revealed increased expression of 14‐3‐3 proteins (and specifically the σ isoform) in all these patients. These results provide novel evidence for the involvement of members of the 14‐3‐3 family of proteins in renal pathology.

The family of 14‐3‐3 proteins consists of small, acidic polypeptides that are present in all eukaryotic cells^26^; in mammals, it consists of 7 members, also known as isoforms (β, γ, ε, η, ζ, σ and τ/θ) exhibiting very high sequence homology.^27^ They bind, acting as docking components, to phosphoserine/phosphothreonine residues of their “client” proteins and via this interaction they contribute to the activity, stability and localization of their binding partners.^11^, ^28^ Due to these characteristics, they are involved in a constellation of cellular processes, such as signal transduction, cell cycle, proliferation, differentiation, apoptosis, metabolism, autophagy,^11^, ^28^, ^29^ acting mainly intracellularly but also extracellularly.^30^ Being involved in so many crucial cellular activities (many of them exhibiting tissue specificity), it is expected that members of the 14‐3‐3 family will have important contributions in several pathological processes and will constitute targets of choice for a variety of pharmaceutical interventions.^31^

Our data suggested that 14‐3‐3σ was the member of the family exhibiting the more dramatic changes in expression in our models of renal pathology. 14‐3‐3σ was originally described as a member with almost exclusive distribution in squamous epithelial tissues^32^; no major expression was observed in the renal parenchyma. In structural terms, 14‐3‐3σ differs from all the other 6 members of the family in the peptide stretch of amino acids 202‐206, where Ile (at position 202), Glu (at 204) and Asp (at 206) are replaced by Met, Asp and His, respectively, in all the other members^27^; these changes might be responsible for any specific structural/functional characteristics of 14‐3‐3σ, as has been suggested for the process of homodimerization.^33^

14‐3‐3σ has been extensively studied in human cancer. It has been suggested that it may act as a tumour suppressor, leading to inhibition of cell cycle progression; as a result cells undergo differentiation and are induced to leave the stem cell compartment.^34^ This effect is thought to be due to its induction by the p53 tumour suppressor protein in response to DNA damage, a property unique to the sigma isoform.^35^ However, although 14‐3‐3σ may exhibit reduced expression in certain cancers, in other cases its expression is increased and in these cases there is resistance to anticancer agents and radiation therapy, thus complicating the situation.^27^

In term of studies regarding the 14‐3‐3σ isoform in kidneys, not much is known, except for its involvement in renal cell carcinoma; cell culture studies demonstrated that when a specific mutant Von Hippel‐Lindau protein is expressed, 14‐3‐3σ is down‐regulated.^36^ However, no other studies exist on the role of 14‐3‐3σ in other pathological processes in the kidney, like renal fibrosis.

In this report, we provide evidence that the family of 14‐3‐3 proteins is increased in abundance in pathological processes other than cancer in the renal parenchyma and we focus on 14‐3‐3σ as a key player in these processes. The up‐regulation of calreticulin was the starting point and we provide evidence that the expression of most members of the family is affected by the levels of calreticulin. This observation is reinforced by our finding that upon calreticulin down‐regulation, the expression of the family of 14‐3‐3 proteins is also decreased both in vitro and in vivo (Figure S5, A‐C).

Although the exact mechanism for this phenomenon is not known, we speculate that it may be mediated by the transcription factor HIF1α, since there is recent evidence from a neuroblastoma cell line that the HIF1α transcript is up‐regulated or down‐regulated following calreticulin up‐regulation or down‐regulation.^37^ We have confirmed this finding in our renal tubular epithelial cell line, where we have observed a 10‐fold increase in the HIF1α transcript when calreticulin is overexpressed (data not shown). As in all the animal models used, hypoxia is a common denominator,^23^, ^24^ we studied the association of the hypoxia‐induced transcription factor HIF1α with the promoter of the gene coding for 14‐3‐3σ and we confirmed specific binding to consensus elements on this promoter. Interestingly, comparison of the acceptor sites for HIF1α between the mouse and human sequences indicated that although the overall identity of the promoters (−2000 bp to +1000 bp) is 64%, and the sequence identity for loci Α, B, C, D and E is ranging between 51 and 90%, in all 5 loci the acceptor sequence for HIF1α is totally conserved between mouse and human promoters. Therefore, our studies, combined with the studies of other groups, suggest that calreticulin overexpression leads to HIF1α up‐regulation which in turn induces the expression of 14‐3‐3σ.

Our data were generated originally in a cell culture system and were then validated in 3 animal models of renal injury each having a different cause/mechanism and damaging different compartments of the kidney. More importantly, the results were also confirmed in 2 different cohorts of patients, suffering from IgA nephropathy and membranous nephropathy. These findings raise the possibility that the expression of 14‐3‐3 proteins and especially 14‐3‐3σ may be used in the future in diagnostic and prognostic tests monitoring the renal parenchyma.

The interactome of 14‐3‐3σ has been studied and 117 binding partners were characterized^38^; in addition, in this study, the largest class of partners was proteins involved in cytoskeletal dynamics. A major class of such proteins are the family of keratins, which are crucial for contributing mechanical stiffness to cells but also components involved in intracellular signalling.^39^ More specifically, keratins 8 and 18 are phosphorylated by Raf1 kinase and in this interaction the involvement of 14‐3‐3 family is crucial for further signalling by keratin heterodimers.^40^ These observations provide a mechanistic explanation for the recent findings that in renal TECs the major keratins (keratins 8 and 18) are up‐regulated and hyper‐phosphorylated in several animal models of renal diseases as well as in patients suffering from renal diseases.^41^

In conclusion, our studies provide evidence for a likely role of 14‐3‐3 family of proteins, especially 14‐3‐3σ, in renal pathology and point out to a possible regulatory mechanism of 14‐3‐3σ gene expression in these phenomena which involves calreticulin and HIF1α.

## CONFLICT OF INTEREST

The authors declare that they have no conflicts of interest.
