# Supplementary material for: The family of 14‐3‐3 proteins and specifically 14‐3‐3σ are up‐regulated during the development of renal pathologies
Source: J Cell Mol Med. 2018 Jun 28;22(9):4139–49. doi: 10.1111/jcmm.13691 (PMC6111864; doi:10.1111/jcmm.13691)

Control A

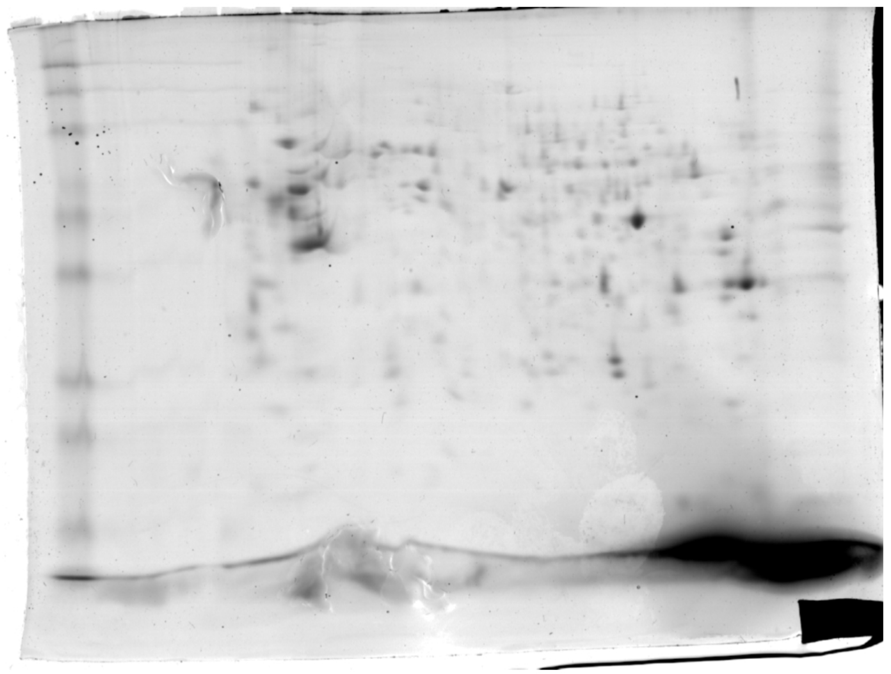

Control B

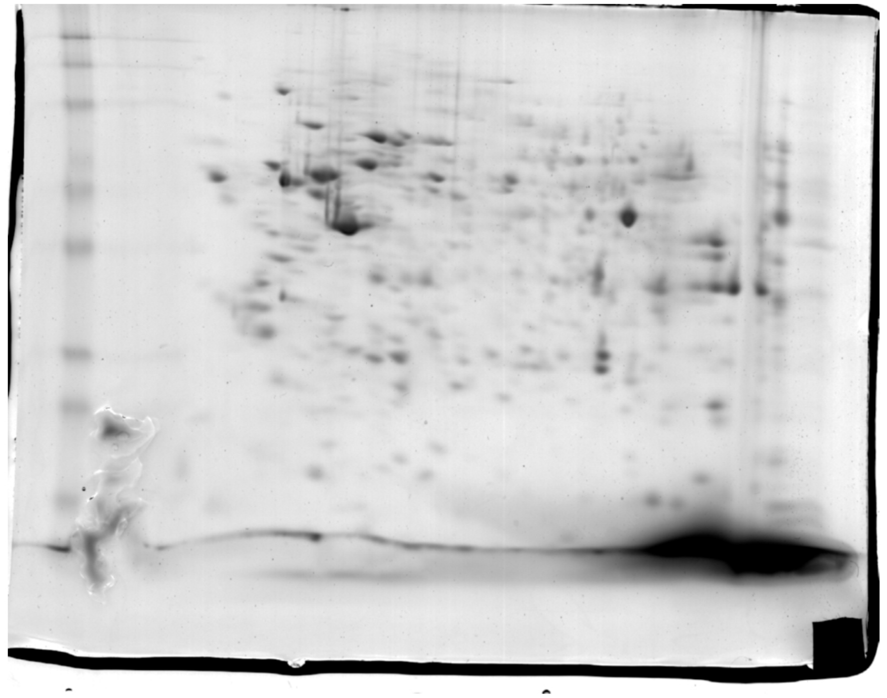

Overexpression A

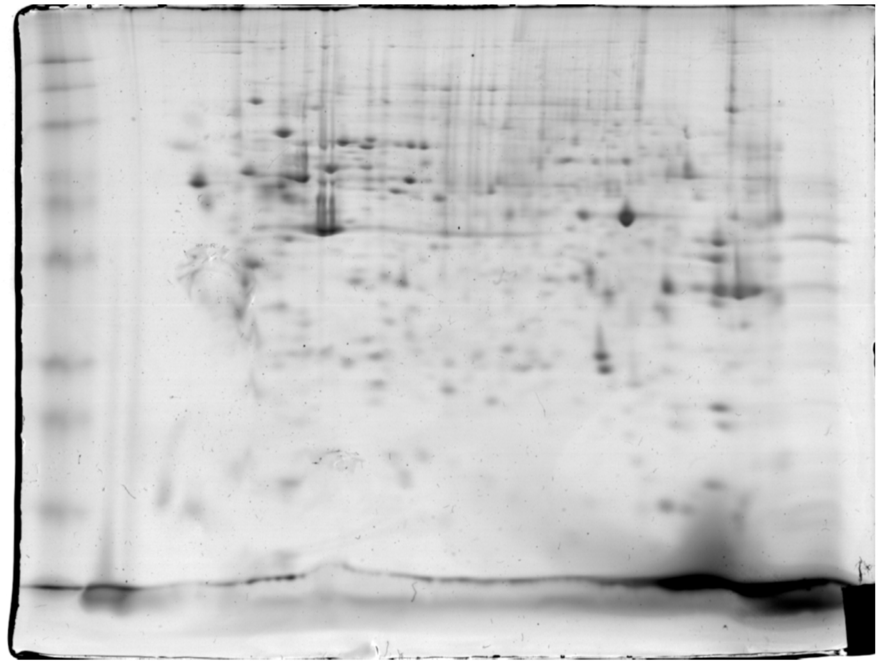

Overexpression B

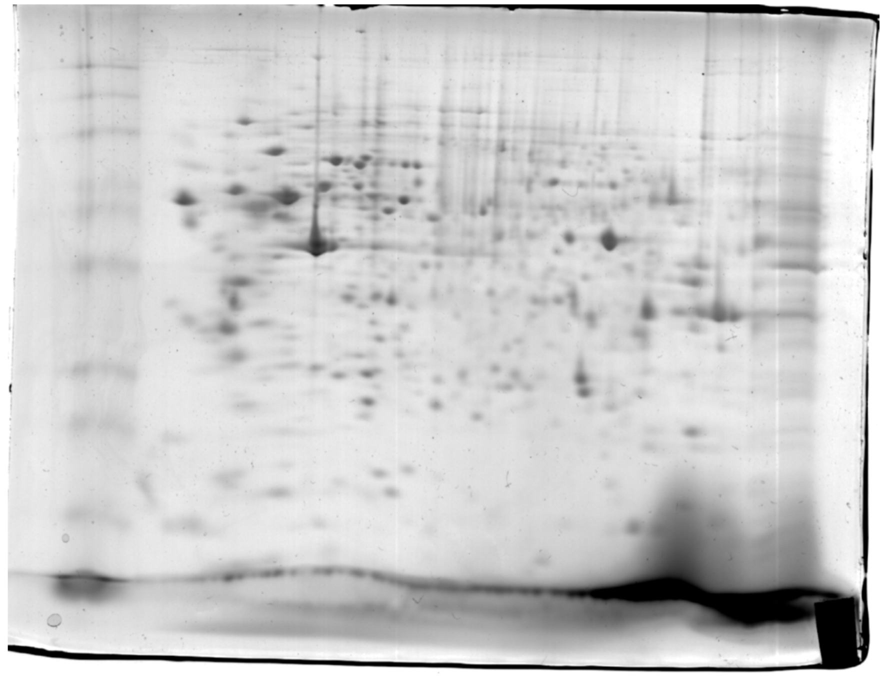

Control A repeat 2

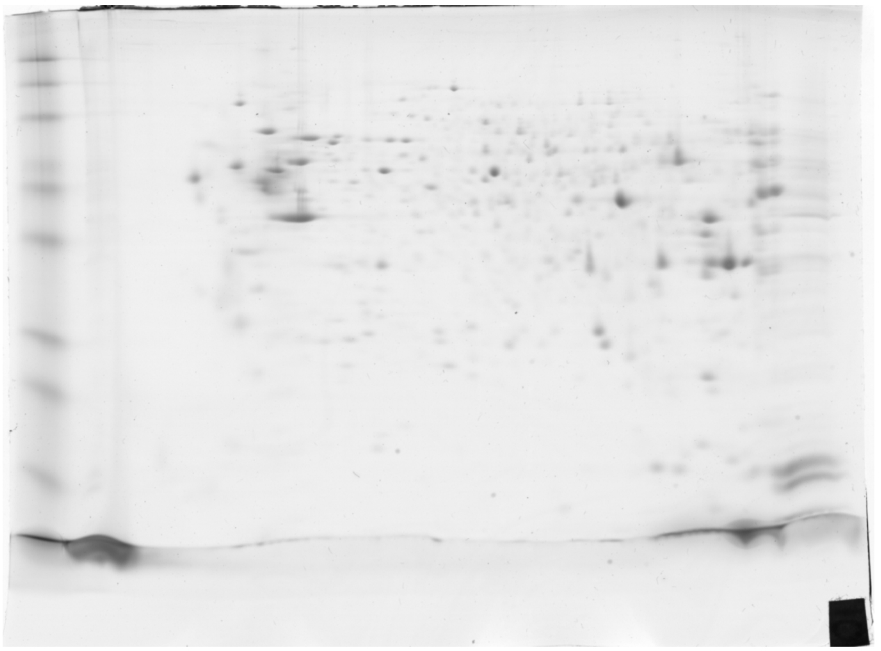

Control B repeat 2

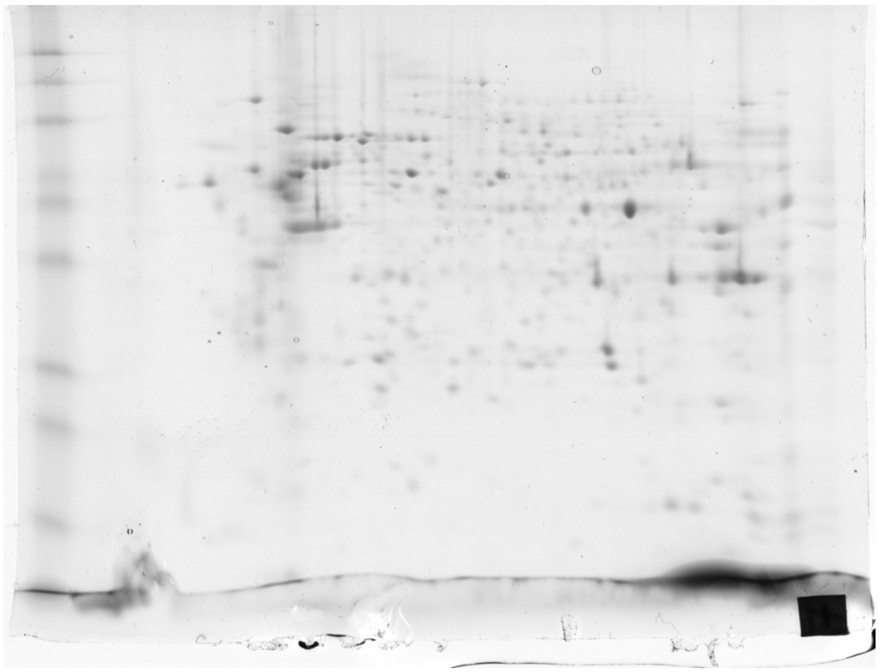

Overexpression A repeat 2

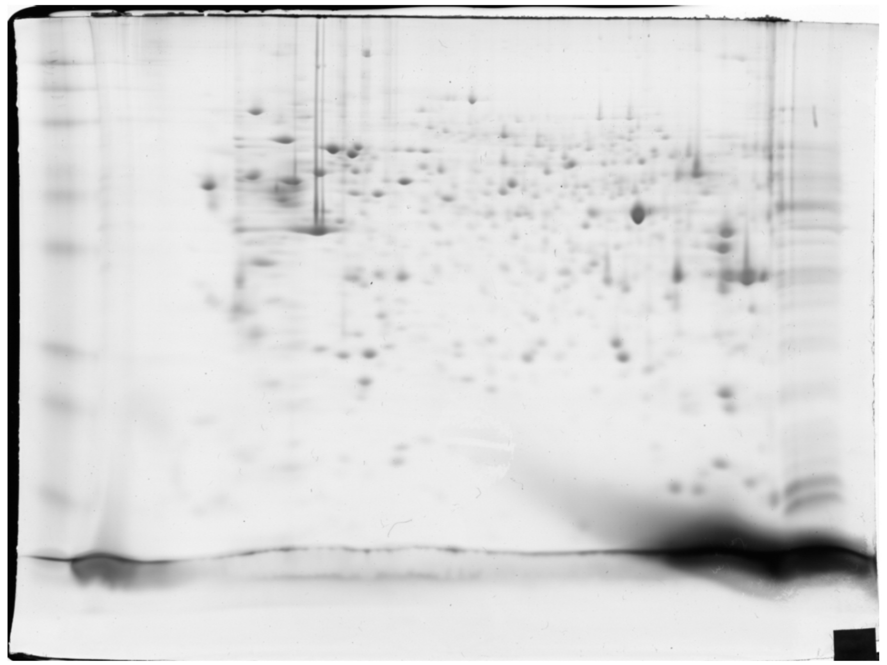

Overexpression B repeat 2

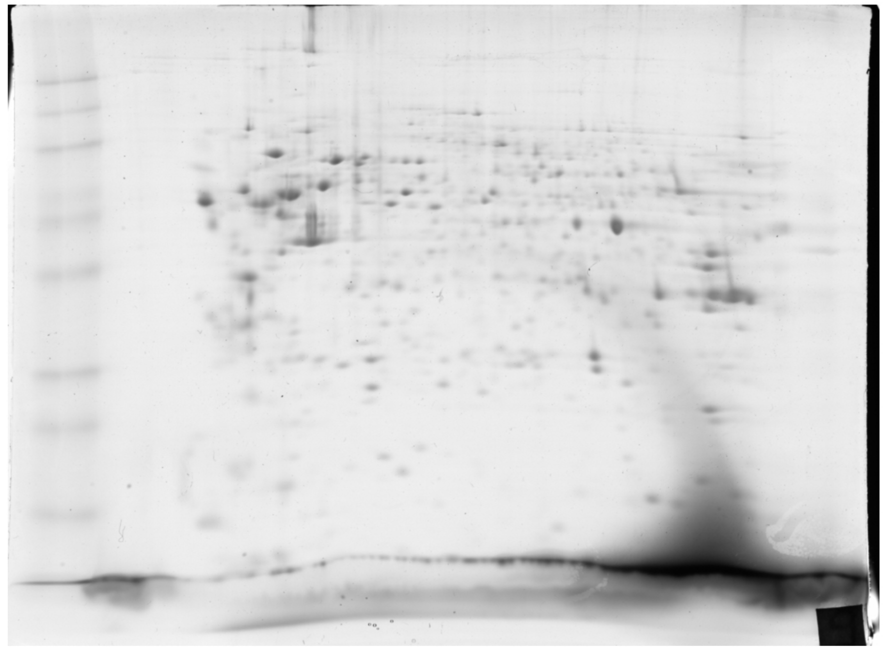

Supplement: Supplementary file 1 [file JCMM-22-4139-s001.pdf]
