# Supplementary material for: The family of 14‐3‐3 proteins and specifically 14‐3‐3σ are up‐regulated during the development of renal pathologies
Source: J Cell Mol Med. 2018 Jun 28;22(9):4139–49. doi: 10.1111/jcmm.13691 (PMC6111864; doi:10.1111/jcmm.13691)
Supplement: Supplementary file 2 [file JCMM-22-4139-s002.pdf]

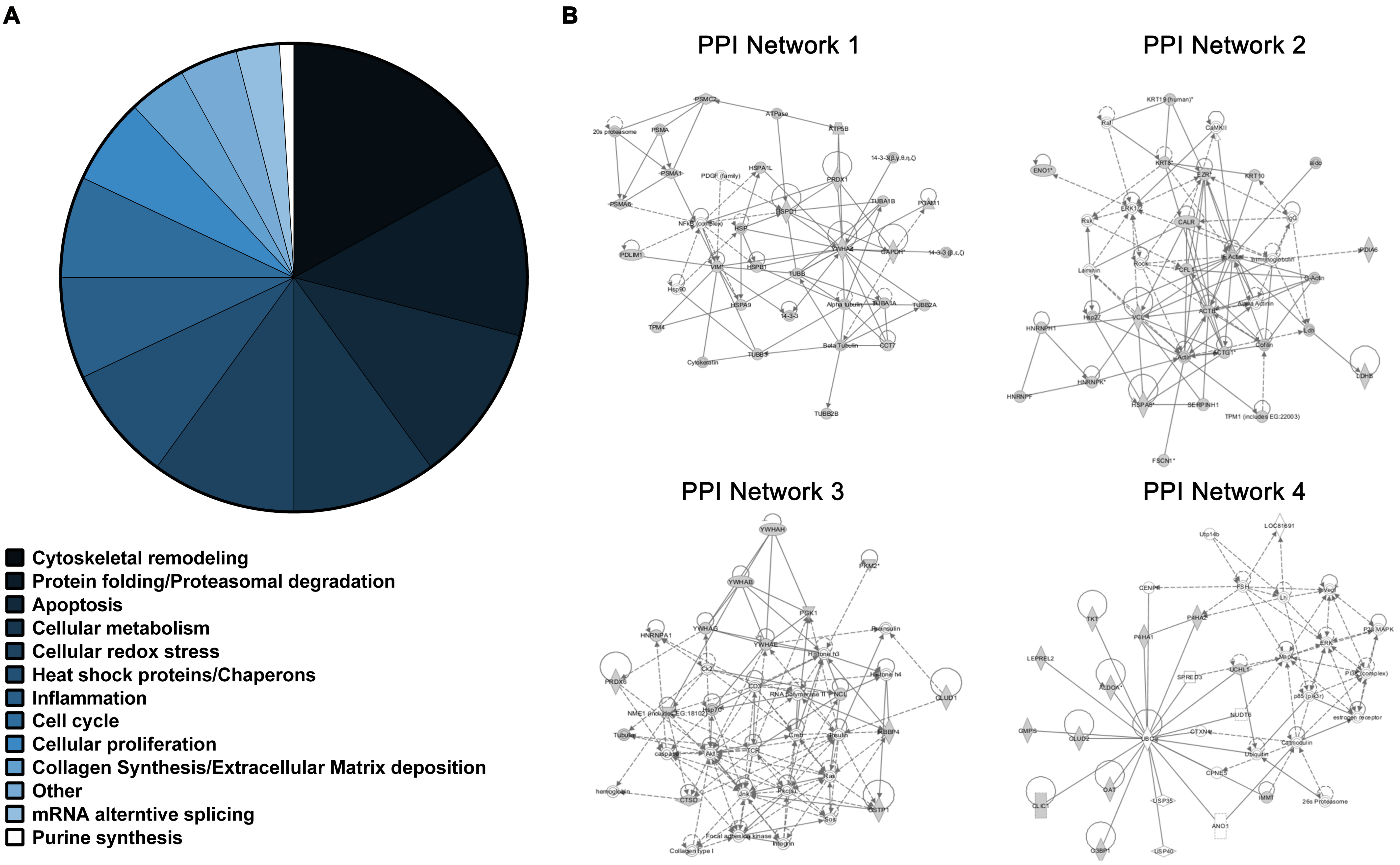
A
B
PPI Network 1
PPI Network 2
PPI Network 3
PPI Network 4
Cytoskeletal remodeling
Protein folding/Proteasomal degradation
Apoptosis
Cellular metabolism
Cellular redox stress
Heat shock proteins/Chaperons
Inflammation
Cell cycle
Cellular proliferation
Collagen Synthesis/Extracellular Matrix deposition
Other
mRNA alterntive splicing
Purine synthesis
