# Supplementary figures and images for: The family of 14‐3‐3 proteins and specifically 14‐3‐3σ are up‐regulated during the development of renal pathologies
Source: J Cell Mol Med. 2018 Jun 28;22(9):4139–49. doi: 10.1111/jcmm.13691 (PMC6111864; doi:10.1111/jcmm.13691)

# Calreticulin and 14-3-3 isoforms mRNA expression

**A**

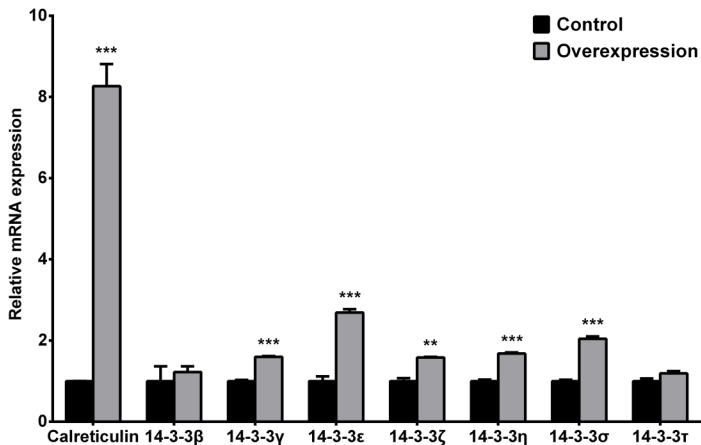

**B**

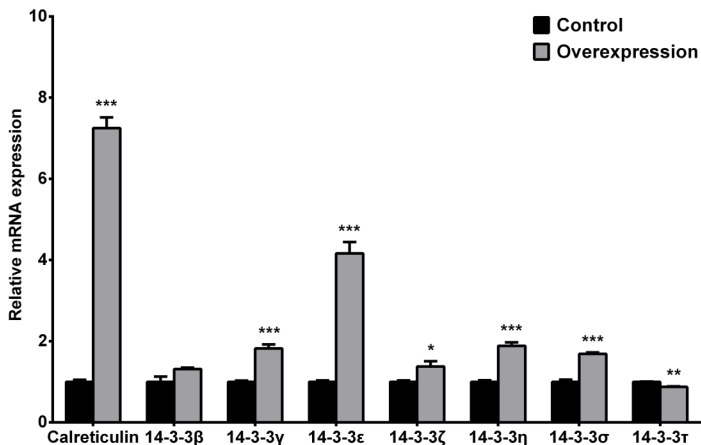

Supplement: Supplementary file 3 [file JCMM-22-4139-s003.pdf]
