# Supplementary material for: The family of 14‐3‐3 proteins and specifically 14‐3‐3σ are up‐regulated during the development of renal pathologies
Source: J Cell Mol Med. 2018 Jun 28;22(9):4139–49. doi: 10.1111/jcmm.13691 (PMC6111864; doi:10.1111/jcmm.13691)

14-3-3 isoforms mRNA expression in UUO model

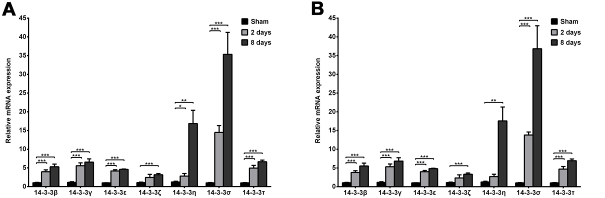

14-3-3 isoforms mRNA expression in NTS model

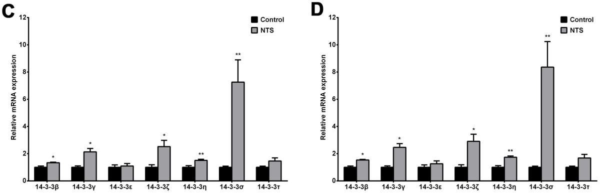

14-3-3 isoforms mRNA expression in IR model

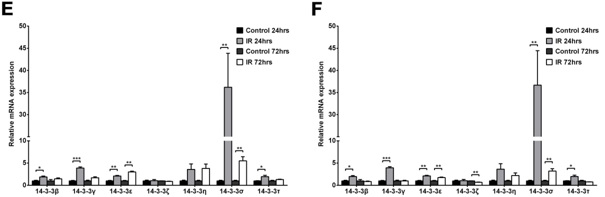

Supplement: Supplementary file 4 [file JCMM-22-4139-s004.pdf]
