# Supplementary material for: The family of 14‐3‐3 proteins and specifically 14‐3‐3σ are up‐regulated during the development of renal pathologies
Source: J Cell Mol Med. 2018 Jun 28;22(9):4139–49. doi: 10.1111/jcmm.13691 (PMC6111864; doi:10.1111/jcmm.13691)
Supplement: Supplementary file 5 [file JCMM-22-4139-s005.pdf]

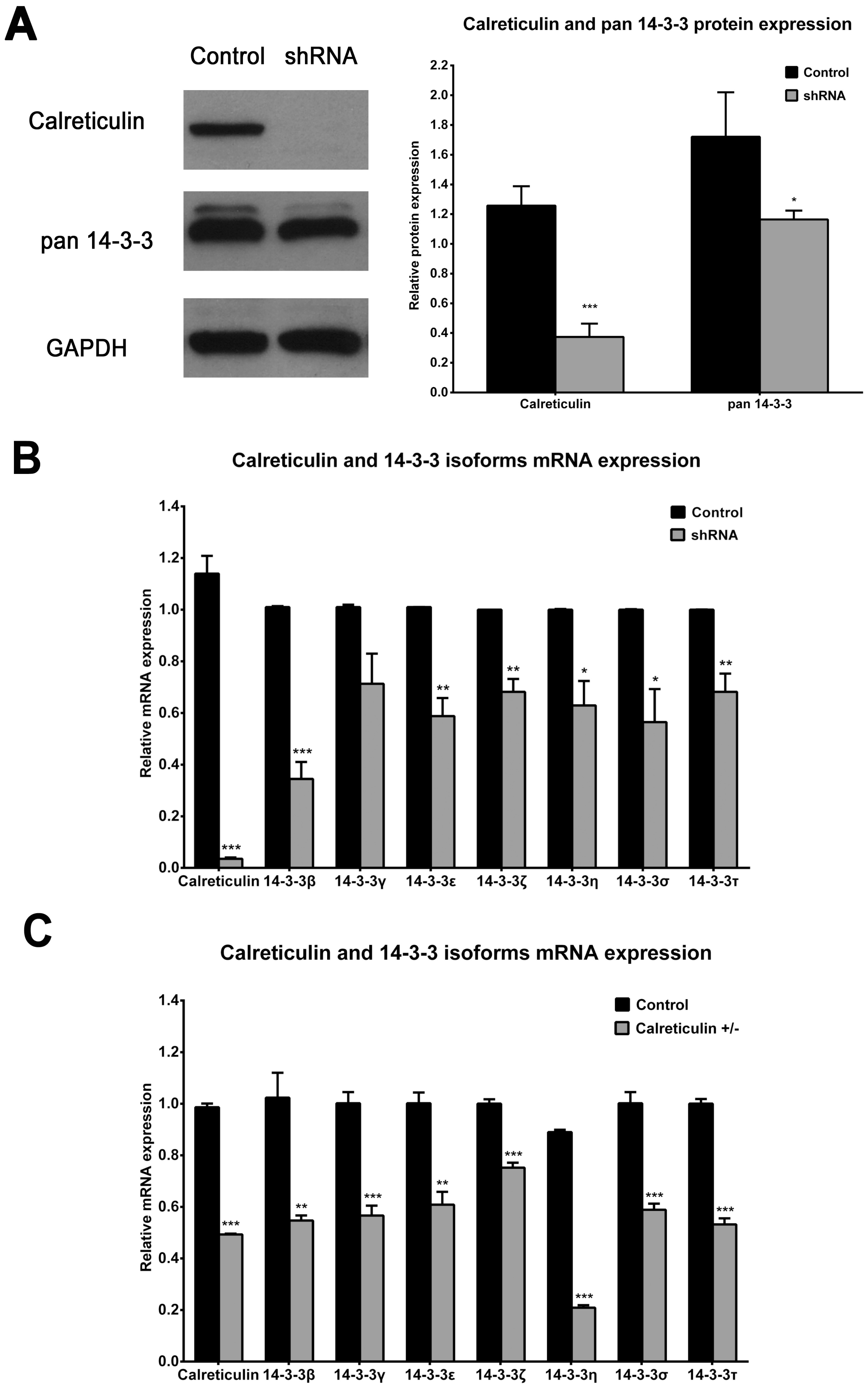

A
Control
shRNA
Calreticulin
pan 14-3-3
GAPDH
Calreticulin and pan 14-3-3 protein expression
Relative protein expression
Control
shRNA
Calreticulin
pan 14-3-3
***
*
B
Calreticulin and 14-3-3 isoforms mRNA expression
Relative mRNA expression
Control
shRNA
Calreticulin
14-3-3β
14-3-3γ
14-3-3ε
14-3-3ζ
14-3-3η
14-3-3σ
14-3-3τ
C
Calreticulin and 14-3-3 isoforms mRNA expression
Relative mRNA expression
Control
Calreticulin +/-
Calreticulin
14-3-3β
14-3-3γ
14-3-3ε
14-3-3ζ
14-3-3η
14-3-3σ
14-3-3τ
