# Supplementary material for: The family of 14‐3‐3 proteins and specifically 14‐3‐3σ are up‐regulated during the development of renal pathologies
Source: J Cell Mol Med. 2018 Jun 28;22(9):4139–49. doi: 10.1111/jcmm.13691 (PMC6111864; doi:10.1111/jcmm.13691)
Supplement: Supplementary file 6 [file JCMM-22-4139-s006.docx]

**Supplementary Table 1:** Mouse primers used for RT-qPCR experiments.

| **Gene** | **Primers** |
| --- | --- |
| 14-3-3β | FW: 5’- ACGGCATTTGATGAGGCGAT - 3’  RV: 5’ - ACGTCCACAGGGTGAGATTG - 3’ |
| 14-3-3γ | FW: 5’- TTCAAGGCGGTCTTCGGTTT - 3’  RV: 5’ - CAGCTCGGTCACGTTCTTCA - 3’ |
| 14-3-3ε | FW: 5’- AGACGCTATCCGCTTCCATC - 3’  RV: 5’ - TCCACCATTTCGTCGTATCGC - 3’ |
| 14-3-3ζ | FW: 5’- TTGAGCAGAAGACGGAAGGT - 3’  RV: 5’ - GAAGCATTGGGGATCAAGAA - 3’ |
| 14-3-3η | FW: 5’- GAAGGCGGTGACAGAGCTGAAT - 3’  RV: 5’ - TGCCATGGTTTTCTGCTCAATG - 3’ |
| 14-3-3σ | FW: 5’- TGGCCCTGAACTTTTCAGTC - 3’  RV: 5’ - GAGGGTGCTGTCCTTGTAGG - 3’ |
| 14-3-3τ | FW: 5’- AAGGAAGCCCATTCGTGTGT - 3’  RV: 5’ - ACACCAGGAAGGCGGAGATA - 3’ |
| HIF1a | FW: 5’- AGGATGAGTTCTGAACGTCGAAA- 3’  RV: 5’ - GGGGAAGTGGCAACTGATGA - 3’ |
| HPRT | FW: 5’- GGAGCGGTAGCACCTCCT - 3’  RV: 5’ - CTGGTTCATCATCGCTAATCAC - 3’ |
| RPL32 | FW: 5’- GCTGCCATCTGTTTTACGG - 3’  RV: 5’ - TGACTGGTGCCTGATGAACT - 3’ |
| 18s | FW: 5’- GCATGCACTCTCCCGTTC - 3’  RV: 5’ - AGCGCGAGAGAGGAGGAG - 3’ |
| GUSB | FW: 5’- CTCTGGTGGCCTTACCTGAT - 3’  RV: 5’ - CAGTTGTTGTCACCTTCACCTC - 3’ |
| GAPDH | FW: 5’- AGGCCGGTGCTGAGTATGTC - 3’  RV: 5’ - TGCCTGCTTCACCACCTTCT - 3’ |
