# Supplementary material for: The family of 14‐3‐3 proteins and specifically 14‐3‐3σ are up‐regulated during the development of renal pathologies
Source: J Cell Mol Med. 2018 Jun 28;22(9):4139–49. doi: 10.1111/jcmm.13691 (PMC6111864; doi:10.1111/jcmm.13691)
Supplement: Supplementary file 7 [file JCMM-22-4139-s007.docx]

**Supplementary Table 2**: Human primers used for RT-qPCR experiments.

| **Gene** | **Primers** |
| --- | --- |
| 14-3-3β | FW: 5’- AGCCTGGCAAAAACGGCATTTGA - 3’  RV: 5’ - GCGTCTCCTTCGTCTCCCTGGT - 3’ |
| 14-3-3γ | FW: 5’- GCGGCCATGAAGAACGTGACAGAG - 3’  RV: 5’ - ATGACCCTCCAGGAAGAGCGGC - 3’ |
| 14-3-3ε | FW: 5’- GCCGAGCAGGCTGAGCGATA - 3’  RV: 5’ - CAGCTCCACATCCATCCCTGCT - 3’ |
| 14-3-3ζ | FW: 5’- CCGCTGGTGATGACAAGAAAGGGAT - 3’  RV: 5’ - AGGGCCAGACCCAGTCTGATAGGA - 3’ |
| 14-3-3η | FW: 5’- CGACGACATGGCCTCCGCTA - 3’  RV: 5’ - CGCCTGGCACCAACCACATT - 3’ |
| 14-3-3σ | FW: 5’- CTGGACAGCCACCTCATCAA - 3’  RV: 5’ - GACCGGGCTGAGCAATGAT - 3’ |
| 14-3-3τ | FW: 5’- ACCTTGCTGAAGTTGCGTGTGGT - 3’  RV: 5’ - CCCCAGGCGGATTGGGTGTG - 3’ |
| Calreticulin | FW: 5’- GCTGGATCGAATCCAAACAC - 3’  RV: 5’ - GAGCATAAAAGCGTGCATCC - 3’ |
| RPL32 | FW: 5’- GTTACGACCCATCAGCCCTTG - 3’  RV: 5’- CATGATGCCGAGAAGGAGATGG - 3’ |
| GUSB | FW: 5’- GAGTGCAAGGAGCTGGACG - 3’  RV: 5’- TGGGGCCTGACTCCCACA - 3’ |
| GAPDH | FW: 5’- GCACCACCAACTGCTTAG - 3’  RV: 5’ - GCCATCCACAGTCTTCTG - 3’ |
