# Supplementary material for: The family of 14‐3‐3 proteins and specifically 14‐3‐3σ are up‐regulated during the development of renal pathologies
Source: J Cell Mol Med. 2018 Jun 28;22(9):4139–49. doi: 10.1111/jcmm.13691 (PMC6111864; doi:10.1111/jcmm.13691)
Supplement: Supplementary file 8 [file JCMM-22-4139-s008.docx]

**Supplementary Table 3**: Specific primers designed for selected sites in mouse 14-3-3σ promoter.

| **Promoter region** | **Primers** |
| --- | --- |
| Locus A  -1811/-1694 | FW: 5’- GCAAAGCCAGTCACCAACTG - 3’  RV: 5’ - GGCACAGGCACAGAACTACA - 3’ |
| Locus B  -1625/-1493 | FW: 5’- TTAGCCAGGACCCTGAAGTC - 3’  RV: 5’ - GCCTGTCTCCTCACAGTGTC - 3’ |
| Locus C  -1140/-1039 | FW: 5’- CTTTCCCTCAGCCCTGTCTT - 3’  RV: 5’ - TCTGTCAGAGGCTGGGAAG - 3’ |
| Locus D  -325/-219 | FW: 5’- ATGCAGCCAGCCCTGAAC - 3’  RV: 5’ - GCTGTGAGTCACCAGGACAGT - 3’ |
| Locus E  +45/+157 | FW: 5’- GCCGAACGGTATGAAGACAT - 3’  RV: 5’ - CCCACCACGTTCTTGTAAGC - 3’ |
