# Supplementary material for: The family of 14‐3‐3 proteins and specifically 14‐3‐3σ are up‐regulated during the development of renal pathologies
Source: J Cell Mol Med. 2018 Jun 28;22(9):4139–49. doi: 10.1111/jcmm.13691 (PMC6111864; doi:10.1111/jcmm.13691)
Supplement: Supplementary file 9 [file JCMM-22-4139-s009.docx]

**Supplementary Table 1:** Identified proteins from differentially expressed protein spots between control cell lines and cell lines that overexpress calreticulin. *Ratio: Intensity of spot from Overexpression cell line A / Intensity of spot from Control cell line A, **Ratio: Intensity of spot from Overexpression cell line B / Intensity of spot from Control cell line B. ND: not determined

| **PROTEIN SPOT** | **PROTEIN NAME** | **ACCESION NUMBER** | **MASCOT SCORE** | **MS COVERAGE** | **THEORETICAL MW** | **pI * **** |
| --- | --- | --- | --- | --- | --- | --- |
| 1 | 14-3-3 protein epsilon | 1433E_HUMAN | 124 | 72 | 29326 | 4.50 2.44 1.44 |
| 2 | 14-3-3 protein zeta/delta | 1433Z_HUMAN | 116 | 67 | 27899 | 4.60 3.46 1.71 |
|  | 14-3-3 protein beta/alpha | 1433B_HUMAN | 86 | 54 | 28179 | 4.61 |
|  | 14-3-3 protein gamma | 1433G_HUMAN | 83 | 60 | 28456 | 4.65 |
|  | 14-3-3 protein eta | 1433F_HUMAN | 58 | 35 | 28372 | 4.61 |
| 3 | Histone-binding protein RBBP4 | RBBP4_HUMAN | 58 | 30 | 47911 | 4.60 2.1 0.96 |
| 4 | Keratin, type I cytoskeletal 19 | K1C19_HUMAN | 259 | 76 | 44079 | 4.90 0.77 1.01 |
| 5 | ATP synthase subunit beta, mitochondrial | ATPB_HUMAN | 162 | 67 | 56525 | 5.10 0.41 0.76 |
|  | Protein disulfide-isomerase A6 | PDIA6_HUMAN | 110 | 52 | 48490 | 4.81 |
| 6 | Tubulin beta chain | TBB5_HUMAN | 228 | 68 | 50095 | 4.60 2.66 3.37 |
| 7 | Vimentin | VIME_HUMAN | 368 | 79 | 53676 | 4.90 0.86 0.95 |
| 8 | Vimentin | VIME_HUMAN | 156 | 56 | 53676 | 4.90 2.49 0.5 |
| 9 | Actin, cytoplasmic 2 | ACTG_HUMAN | 74 | 34 | 42108 | 5.20 ND ND |
| 10 | Chloride intracellular channel protein 1 | CLIC1_HUMAN | 94 | 47 | 27248 | 4.90 0.7 0.67 |
| 11 | Actin, cytoplasmic 1 | ACTB_HUMAN | 139 | 49 | 42052 | 5.20 3.29 2.07 |
| 12 | Actin, cytoplasmic 1 | ACTB_HUMAN | 134 | 57 | 42052 | 5.20 0.14 0.85 |
| 13 | Actin, cytoplasmic 1 | ACTB_HUMAN | 117 | 43 | 42052 | 5.20 ND ND |
| 14 | Vimentin | VIME_HUMAN | 166 | 59 | 53676 | 4.90 |
| 15 | Actin, cytoplasmic 1 | ACTB_HUMAN | 89 | 48 | 42052 | 5.20 0.08 0.95 |
| 16 | Actin, cytoplasmic 1 | ACTB_HUMAN | 105 | 42 | 42052 | 5.20 1.02 0.31 |
| 17 | Actin, cytoplasmic 1 | ACTB_HUMAN | 58 | 33 | 42052 | 5.20 |
| 18 | Actin, cytoplasmic 1 | ACTB_HUMAN | 91 | 39 | 42052 | 5.20 1.29 1.00 |
| 19 | Vimentin | VIME_HUMAN | 130 | 56 | 53676 | 4.90 1.61 1.32 |
| 20 | Heterogeneous nuclear ribonucleoprotein K | HNRPK_HUMAN | 72 | 37 | 51230 | 5.30 0.45 0.33 |
| 21 | Ras GTPase-activating protein-binding protein 1 | G3BP1_HUMAN | 63 | 34 | 52189 | 5.30 1.73 0.98 |
| 22 | Heat shock cognate 71 kDa protein | HSP7C_HUMAN | 90 | 35 | 71082 | 5.20 1.27 0.26 |
| 23 | Heat shock cognate 71 kDa protein | HSP7C_HUMAN | 131 | 36 | 71082 | 5.20 0.6 0.61 |
| 24 | Actin, cytoplasmic 1 | ACTB_HUMAN | 67 | 39 | 42052 | 5.20 1.55 1.55 |
| 25 | Ubiquitin carboxyl-terminal hydrolase isozyme L1 | UCHL1_HUMAN | 111 | 59 | 25151 | 5.20 0.66 0.67 |
| 26 | Actin, cytoplasmic 1 | ACTB_HUMAN | 57 | 38 | 42052 | 5.20 1.63 0.15 |
| 27 | Tubulin alpha-1B chain | TBA1B_HUMAN | 63 | 35 | 50804 | 4.80 0.95 ND. |
| 28 | Keratin, type II cytoskeletal 8 | K2C8_HUMAN | 61 | 23 | 53671 | 5.40 1.13 0.21 |
| 29 | Heat shock 70 kDa protein 1A/1B | HSP71_HUMAN | 235 | 58 | 70294 | 5.40 1.17 2.28 |
| 30 | Prolyl 4-hydroxylase subunit alpha-2 | P4HA2_HUMAN | 78 | 29 | 61263 | 5.40 1.16 0.63 |
| 31 | Heat shock 70 kDa protein 1A/1B | HSP71_HUMAN | 91 | 34 | 70294 | 5.40 1.82 1.63 |
|  | Heat shock cognate 71 kDa protein | HSP7C_HUMAN | 66 | 30 | 71082 | 5.24 |
|  | Stress-70 protein, mitochondrial | GRP75_HUMAN | 57 | 29 | 73920 | 5.81 |
| 32 | L-lactate dehydrogenase B chain | LDHB_HUMAN | 110 | 55 | 36900 | 5.70 2.39 1.78 |
| 33 | 26S protease regulatory subunit 7 | PRS7_HUMAN | 102 | 40 | 49002 | 5.60 0.48 0.37 |
| 34 | Heterogeneous nuclear ribonucleoprotein H | HNRH1_HUMAN | 55 | 36 | 49484 | 5.90 4.55 4.44 |
| 35 | Mitochondrial inner membrane protein | IMMT_HUMAN | 57 | 20 | 84026 | 6.10 2.07 1.30 |
| 36 | Peroxiredoxin-6 | PRDX6_HUMAN | 90 | 56 | 25133 | 6.00 1.38 0.55 |
| 37 | Proteasome subunit alpha type-1 | PSA1_HUMAN | 61 | 37 | 29822 | 6.20 1.73 2.59 |
| 38 | Ezrin | EZRI_HUMAN | 79 | 29 | 69484 | 5.90 0.39 0.97 |
| 39 | Ezrin | EZRI_HUMAN | 61 | 25 | 69484 | 5.90 ND ND |
| 40 | Prolyl 3-hydroxylase 3 | P3H3_HUMAN | 60 | 23 | 82584 | 5.90 ND ND |
| 41 | Vinculin | VINC_HUMAN | 241 | 45 | 124292 | 5.40 1.43 2.58 |
| 42 | Proteasome subunit alpha type-6 | PSA6_HUMAN | 52 | 32 | 27838 | 6.40 0.89 1.96 |
| 43 | Phosphoglycerate mutase 1 | PGAM1_HUMAN | 89 | 59 | 28900 | 6.80 0.7 0.55 |
| 44 | PDZ and LIM domain protein 1 | PDLI1_HUMAN | 85 | 55 | 36505 | 6.60 1.51 1.46 |
| 45 | GMP synthase [glutamine-hydrolyzing] | GUAA_HUMAN | 102 | 34 | 77408 | 6.40 0.89 0.55 |
| 46 | Alpha-enolase | ENOA_HUMAN | 211 | 61 | 47481 | 7.70 1.96 2.27 |
| 47 | Keratin, type I cytoskeletal 10 | K1C10_HUMAN | 52 | 26 | 59020 | 5.00 0.31 0.21 |
| 48 | Fascin | FSCN1_HUMAN | 107 | 44 | 55123 | 7.00 0.43 0.21 |
| 49 | Glutamate dehydrogenase 1, mitochondrial | DHE3_HUMAN | 153 | 50 | 61701 | 8.50 0.78 1.41 |
|  | Fascin | FSCN1_HUMAN | 58 | 36 | 55123 | 7.02 |
| 50 | Peroxiredoxin-1 | PRDX1_HUMAN | 96 | 68 | 22324 | 9.20 1.56 1.32 |
| 51 | Fructose-bisphosphate aldolase A | ALDOA_HUMAN | 122 | 53 | 39851 | 9.20 1.35 1.37 |
| 52 | Alpha-enolase | ENOA_HUMAN | 176 | 66 | 47481 | 7.70 2.34 5.12 |
| 53 | Pyruvate kinase isozymes M1/M2 | KPYM_HUMAN | 68 | 28 | 58470 | 9.00 0.24 0.08 |
| 54 | T-complex protein 1 subunit eta | TCPH_HUMAN | 76 | 32 | 59842 | 8.60 0.33 0.29 |
| 55 | Pyruvate kinase isozymes M1/M2 | KPYM_HUMAN | 271 | 65 | 58470 | 9.00 1.44 1.69 |
| 56 | Pyruvate kinase isozymes M1/M2 | KPYM_HUMAN | 169 | 57 | 58470 | 9.00 1.16 2.19 |
|  | Transketolase | TKT_HUMAN | 80 | 39 | 68519 | 8.54 |
| 57 | Cofilin-1 | COF1_HUMAN | 78 | 59 | 18719 | 9.10 0.65 1.76 |
| 58 | Glyceraldehyde-3-phosphate dehydrogenase | G3P_HUMAN | 138 | 52 | 36201 | 9.30 1.23 0.32 |
| 59 | Fructose-bisphosphate aldolase A | ALDOA_HUMAN | 129 | 65 | 39851 | 9.20 0.3 0.2 |
| 60 | Glyceraldehyde-3-phosphate dehydrogenase | G3P_HUMAN | 167 | 60 | 36201 | 9.30 0.95 1.96 |
| 61 | Heterogeneous nuclear ribonucleoprotein A1 | ROA1_HUMAN | 103 | 43 | 38837 | 9.60 2.37 2.74 |
| 62 | Fructose-bisphosphate aldolase A | ALDOA_HUMAN | 189 | 70 | 39851 | 9.20 0.4 0.96 |
| 63 | Serpin H1 | SERPH_HUMAN | 59 | 37 | 46525 | 9.30 0.18 0.73 |
| 64 | Phosphoglycerate kinase 1 | PGK1_HUMAN | 187 | 64 | 44985 | 9.20 0.64 1.14 |
| 65 | Nucleolin | NUCL_HUMAN | 120 | 26 | 76625 | 4.40 |
| 66 | Ezrin | EZRI_HUMAN | 168 | 41 | 69484 | 5.90 0.45 0.6 |
| 67 | 60 kDa heat shock protein, mitochondrial | CH60_HUMAN | 147 | 44 | 61187 | 5.60 2.1 1.66 |
| 68 | Prolyl 4-hydroxylase subunit alpha-1 | P4HA1_HUMAN | 85 | 29 | 61296 | 5.60 |
| 69 | Calreticulin | CALR_HUMAN | 235 | 66 | 48283 | 4.10 1.35 1.62 |
| 70 | Keratin, type II cytoskeletal 8 | K2C8_HUMAN | 175 | 46 | 53671 | 5.40 1.40 1.05 |
| 71 | Ornithine aminotransferase, mitochondrial | OAT_HUMAN | 71 | 23 | 48846 | 6.60 ND ND |
| 72 | Keratin, type I cytoskeletal 19 | K1C19_HUMAN | 290 | 84 | 44079 | 4.90 0.67 0.93 |
| 73 | Tropomyosin alpha-1 chain | TPM1_HUMAN | 59 | 25 | 32746 | 4.50 0.8 0.78 |
| 74 | Tropomyosin alpha-4 chain | TPM4_HUMAN | 76 | 31 | 28619 | 4.50 0.87 0.02 |
| 75 | Heat shock protein beta-1 | HSPB1_HUMAN | 96 | 57 | 22826 | 6.00 0.96 0.57 |
| 76 | Cathepsin D | CATD_HUMAN | 53 | 19 | 45037 | 6.10 1.02 0.52 |
| 77 | Glutathione S-transferase P | GSTP1_HUMAN | 109 | 52 | 23569 | 5.30 0.34 ND |
| 78 | Nucleoside diphosphate kinase A | NDKA_HUMAN | 60 | 42 | 17309 | 5.80 1.05 1.19 |
| 79 | Eukaryotic translation initiation factor 3 subunit I | EIF3I_HUMAN | 95 | 35 | 36878 | 5.30 |
| 80 | Annexin A2 | ANXA2_HUMAN | 138 | 59 | 38808 | 8.50 1.72 0.24 |
| 81 | Tropomyosin alpha-1 chain | TPM1_HUMAN | 50 | 19 | 32746 | 4.50 |
